# Supplementary material for: Regulation of plant immune receptor accumulation through translational repression by a glycine-tyrosine-phenylalanine (GYF) domain protein
Source: eLife. 2017 Mar 31;6:e23684. doi: 10.7554/eLife.23684 (PMC5403212; doi:10.7554/eLife.23684)
Supplement: Supplementary file 1. — DOI: http://dx.doi.org/10.7554/eLife.23684.014 [file elife-23684-supp1.pdf]

Supplementary file 1. Summary of primers used in this study

Primer name	Sequence (5'-3')	Note
92-1-F	CACCACAGTGGCTTCTCTCC	to genotype *muse11-1* mutantions
92-1-wt-R	CAGGACCCCAGCGAGAGTAC	to genotype *muse11-1* mutantions
92-1-mt-R	CAGGACCCCAGCGAGAGTCT	to genotype *muse11-1* mutantions
LK83-wt-F	GGACACCTTGTCCTCAGTGC	to genotype *muse11-2* mutantions
LK83-m-F	GGACACCTTGTCCTCAGTGT	to genotype *muse11-2* mutantions
92-1-R	GGTTCTCCAGGACCACTTTC	to genotype *muse11-2* mutantions
92-2 genomic-KpnI-F	GCGGTACCattggtggtccaatagtagc	to amplify genomic *EXA1* for *EXA1::EXA1-GFP* constuct
92-2 genomic-XbaI-R	GCTCTAGActatcaaagtgtcagccgac	to amplify genomic *EXA1* for *EXA1::EXA1-GFP* constuct
92-3 genomic-XbaI-F	GCTCTAGAggagactcctgcaactgaag	to amplify genomic *EXA1* for *EXA1::EXA1-GFP* constuct
92-4 genomic-SalI-R	GCGTCGACtccccaaattccgagtaagc	to amplify genomic *EXA1* for *EXA1::EXA1-GFP* constuct
MUSE11-KpnI-F	gcgcggtaccATGGCTAACTCTTCCGCTGG	to amplify genomic *EXA1* for *35S::EXA1-FLAG* constuct
MUSE11-SmaI-R	tcccccgggGTCCTCAATTGTCTGAATCT	to amplify genomic *EXA1* for *35S::EXA1-FLAG* constuct
MUSE11-SalI-R	CGCGTCGACTCAGTCCTCAATTGTCTGA	to amplify genomic *EXA1* for *35S::EXA1-Cluc* constuct
RPL18-KpnI-F	gcgcggtaccATGgtacttctctcctcctc	to amplify genomic RPL18 for *35S::RPL18-Nluc* constuct
RPL18-SalI(no stop)-R	CGCGTCGACAACCTTGAATCCACGACTC	to amplify genomic RPL18 for *35S::RPL18-Nluc* constuct
salk_005994-F	TTCGCCTGAAGAACTTTCAC	to genotype *exa1-1* mutantions
salk_005994-R	GCCGCTCAAGTTCCAATTTC	to genotype *exa1-1* mutantions
salk_058114-F	AAACCCATGCTCACTAGCTG	to genotype salk_058114 mutations
salk_058114-R	ATCCCATTCTTCATCATCGC	to genotype salk_058114 mutations
salk_035304-F	CACCATAATAGTTCCTTACT	to genotype salk_035304 mutations
salk_035304-R	ATCCTTCATGCGACGAAGAA	to genotype salk_035304 mutations
eIF4E-KpnI-F	gcgcggtaccATGGCGGTAGAAGACAC	to amplify genomic eIF4E for *35S::eIF4E-Nluc* constuct
eIF4E-SalI(no stop)-R	CGCGTCGACAGCGGTGTAAGCGTTCT	to amplify genomic eIF4E for *35S::eIF4E-Nluc* constuct
RPS2_ real_F3	CATATACAGCATCTCCACGTTG	Real time PCR
RPS2_ real_R3	CACAGCCTTCACAACTTAACCA	Real time PCR
RPS4_real_F2	GCTAGACCATGTCTTCATTGGA	Real time PCR
RPS4 real R2	GTACGTTATGCCTGTTGAAAAGA	Real time PCR
Hygo-real-F	gttgcaagacctgcctg	Real time PCR
Hygp-real-R	GAAATCCGCGTGCACGA	Real time PCR
SNC1 F	CTG GGA TAA GTT GTA TCG TGT TG	Real time PCR
SNC1 R	AGA TGT CCC CGA TGT CAT CCG	Real time PCR
